# MiR-186 Suppressed Growth, Migration, and Invasion of Lung Adenocarcinoma Cells via Targeting Dicer1

**DOI:** 10.1155/2021/6217469

**Published:** 2021-11-11

**Authors:** Juan Wang, Yi Zhang, Fanghong Ge

**Affiliations:** ^1^Department of Oncology, Tongzhou Hospital Affiliated to Nantong University, Nantong, Jiangsu 226300, China; ^2^Department of Oncology, Rich Hospital Affiliated to Nantong University, Nantong, Jiangsu 226010, China; ^3^Department of Radiotherapy, Cancer Hospital Affiliated to Nantong University, Nantong, Jiangsu 226361, China

## Abstract

**Objective:**

Globally, the fatal form of lung cancer is non-small-cell lung cancer (NSCLC), and its most common subtype is lung adenocarcinoma (LUAD). In cancer development and progression, miRNAs play key roles primarily in interacting with cancer-related genes. The main focus of this research was to examine the biological roles of miR-186 in LUAD.

**Methods:**

We examined tissues of LUAD and lung cancer cell lines. The expressions of miR-186, Dicer1, Ki-67, and PCNA were determined by immunohistochemistry (IHC), real-time quantitative PCR (RT-PCR), and western blot assays. The CCK-8 and transwell assays were used to determine cell proliferation, migration, and invasion. To determine the association between miR-186 and Dicer1, a luciferase assay was used.

**Results:**

MiR-186 expression was found to be lower in LUAD tissues, and this was correlated to TNM stage and lymph node metastasis in LUAD patients. miR-186 upregulation significantly reduced the proliferation rate and the level of Ki67 and PCNA of LUAD cell lines HCC827 and A549. Transwell assay exhibited that miR-186 upregulation considerably reduced HCC827 and A549 cells' migration and invasion abilities. Furthermore, we also confirmed that Dicer1 was a direct target of miR-186. Importantly, Dicer1 overexpression abolished the suppression of miR-186 mimics on cell proliferation, migration, and invasion of HCC827 and A549 cells.

**Conclusion:**

These results indicated that the miR-186/Dicer1 pathway is critical for regulating LUAD cell proliferation, migration, and invasion.

## 1. Introduction

Lung cancer is the most common type of cancer in the world, and non-small-cell lung cancer (NSCLC) is the most prevalent subtype of lung cancer (85%) [1, 2]. Although advancements in diagnosing and treating have been made in recent years, patients with NSCLC still have a dismal five-year survival rate [3, 4]. Lung adenocarcinoma (LUAD) is the most prevalent subtype of NSCLC, accounting for 47% of cases. In comparison, the prevalence of two other prominent subtypes of NSCLC, lung squamous cell carcinoma (LUSC) and large-cell carcinoma, is 35% and 17%, respectively [5]. As a result, it is worthwhile to investigate the molecular pathway through which LUAD develops.

Micro RNAs (small noncoding RNAs) inhibit target genes posttranscriptionally. Previous studies have suggested that miRNAs work critically in regulating cancer progression and development via interacting with cancer-related genes. For instance, miR-597-5p inhibited cell proliferation, lowered relative wound width, restricted colony formation, and downregulated invasive cell population in pancreatic cancer and increased cell death via ELK1 targeting [6]. miR-452 was also overexpressed in gastrointestinal cancer tissues and lines of cell and increased tumor cell proliferation, migration, and S-phase arrest by inhibiting EPB41L3. In the study of Liao et al. [7], the miR-206 was reported to decrease the NSCLC cell proliferation, migration, and invasion by downregulating the CORO1C. Hence, miRNA expression and biological activity in cancers must be studied to help find diagnostic and therapeutic targets for tumors.

In the variety of malignancies, miR-186 has been widely investigated, including LUAD, but its biological functions in lung cancer remain contentious [8]. MiR-186 inhibition had previously been revealed to reduce NSCLC cell proliferation, migration, and invasion [9].

Upregulation of miRNA-186 has been reported to reduce cisplatin resistance of NSCLC cells via targeting SIX1. In a previous study, miR-186 had been reported to reduce the inhibited PMI of NSCLC cells [10]. The inhibition of lung cell proliferation is associated with miR-186 targeting SIRT6 [11]. Moreover, miR-186 downregulation is related to poor survival in LUAD as it mediated the inhibition of cell-cycle progression [12]. In contrast, in the research conducted by Feng et al., miR-186 was found capable of increasing LUAD cell growth, migration, and invasion by targeting PTEN [13]. Therefore, we hypothesized that miR-186 may function as a tumor-suppressor in LUAD.

In our current study, we observed that miR-186 levels were lower in LUAD tissues and were linked to lymph node and TNM stage metastases in LUAD patients. Moreover, the upregulation of miR-186 decreased cell proliferation, migration, and invasion through targeting Dicer1.

## 2. Materials and Methods

### 2.1. Clinical Data

We collected 35 LUAD tumor tissues and corresponding normal tissues at Cancer Hospital Affiliated with Nantong University between February 2017 and February 2019. All patients with LUAD did not undergo radiotherapy or chemotherapy before surgery. The Ethical Committee of the Cancer Hospital Affiliated with Nantong University approved this research, and all patients involved in the study signed the written consent form.

### 2.2. Immunohistochemistry (IHC) Assay

For the IHC assay, formalin was used to fix tumor tissues and normal tissues, and then, tissues were embedded in paraffin. The sections were incubated with Dicer1 antibody (Abcam) overnight at 4 °C. Following incubation with a biotinylated secondary antibody, the sections were stained with hematoxylin. The IHC scores were assessed as previously described [14].

### 2.3. LUAD Cell Lines

Four human cell lines (HCC827 [15], A549 [16], NCI–H23 [17], and NCI–H358 [18]) and the ATCC (Rockville, MD, USA), a standard human lung epithelial cell line BEAS-2B was provided. DMEM, Thermo Fisher, USA, containing 10% fetal bovine serum (FBS, Hyclone, USA) was used to culture A549 and BEAS-2B cells. HCC827, NCI–H23, and NCI–H358 were grown in RPMI-1640 medium containing 10% FBS. All cells were cultured in a humidified atmosphere with 5% CO_2_, at room temperature.

### 2.4. The Extraction of RNA and Real-Time Quantitative PCR (RT-qPCR)

We isolated the total RNA of patient tissue samples or indicated cells by utilizing Trizol reagent (Invitrogen, USA). The PrimeScriptTM RT reagent kit (Takara, Dalian) was used to generate the cDNA (Takara, Dalian), and qPCR was conducted using the SYBR Premix Ex Taq II kit. All specific primers are mentioned as follows:

forward primer (F) 5′-3' and reverse primer (*R*) 5′-3'. miR-186, F : ACACTCCAGCTGGGCAAAGAATTCTCCTTT, R : CTCAACTGGTGTCGTGGAGTCGGCAATTCAGTTGAGAGCCCAAA; Dicer1, F : GAGCTGTCCTATCAGATCAGGG, R : ACTTGTTGAGCAACCTGGTTT; Ki67, F : GGGCCAATCCTGTCGCTTAAT, R : GTTATGCGCTTGCGAACCT; PCNA, F : CCTGCTGGGATATTAGCTCCA, R : CAGCGGTAGGTGTCGAAGC; U6 : F: AAAGCAAATCATCGGACGACC, R : GTACAACACATTGTTTCCTCGGA; and *β*-acti*n*, F : CTTAGTTGCGTTACACCCTTTCTTG, R : CTGTCACCTTCACCGTTCCAGTTT. *U*6 or *β*-actin were considered as endogenous controls.

### 2.5. Cell Transfection

Random RNAs were designed as control mimics and control inhibitors. The mimics and inhibitors for miR-186/control and the Dicer1 cDNA that was cloned into the pcDNA3.1 vector (pcDNA-Dicer1) were designed and manufactured by GenePharma (Shanghai, China). The pcDNA3.1 empty vector was used as a negative control (pcDNA) (GenePharma, Shanghai, China). Lipofectamine 2000 (Invitrogen) was used to transfect the cells.

### 2.6. CCK-8 Assay

Cells (1000 cells per well) were transfected with miR-186 mimics, or pcDNA-Dicer 1 was grown in 96-well plates. After 0, 24, 48, and 72 h, each well was treated with 10 *μ*l solution of CCK-8, and the cells were incubated at 37 °C for 4 hours. The plate reader was used to measure absorbance at 450 nm.

### 2.7. Migration and Invasion Assay

A BD BioCoat Chamber (BD Biosciences) was utilized to observe cell migration and invasion activity. Cells (5 × 10^4^/ml) transfected with miR-186 mimics or pcDNA-Dicer 1 in the growth medium without serum and were seeded into the upper chamber with or without a precoating Matrigel matrix. The lower wells were filled with the complete medium. The penetrated cells were fixed, stained with crystal violet, and counted under microscopy after 24 hours.

### 2.8. Luciferase Assay

The psi-CHECK-2 luciferase reporter vectors (LRVs) with the wild-type (WT) or mutant Dicer 1–3′UTR were generated by GenePharma. Cells were cotransfected with LRV and miR-186 mimics or negative control miRNA. After 24 h of transfection, the dual-luciferase assay kit (Promega) was used to assess luciferase activity.

### 2.9. Western Blot

Total protein was extracted from transfected cells using ProteoJET Mammalian Cell Lysis Reagent (MBI Fermentas) containing a proteinase inhibitor cocktail (Boster, China). Equal amounts of proteins were separated using SDS-PAGE. At 4°C overnight, the membranes were incubated with primary antibodies. Super ECL Plus Detection Reagent (Applygen Technologies Inc., China) was applied for detecting the protein signal. Dicer 1 (Abcam, ab259327, 1 : 1000) and GAPDH (Abcam, ab9485, 1 : 1000) were used as primary antibodies.

### 2.10. Statistical Analysis

All data were presented as the mean ± standard error (SD) and analyzed using Student's *t*-test or one-way ANOVA test between two or more groups. ^*∗*^*P* < 0.05, ^*∗∗*^*P* < 0.01, and ^*∗∗∗*^*P* < 0.001 were considered statistically significant.

## 3. Results

### 3.1. Expressions of miR-186 in LUAD Tissue and Cell Lines

**Figure 1:**
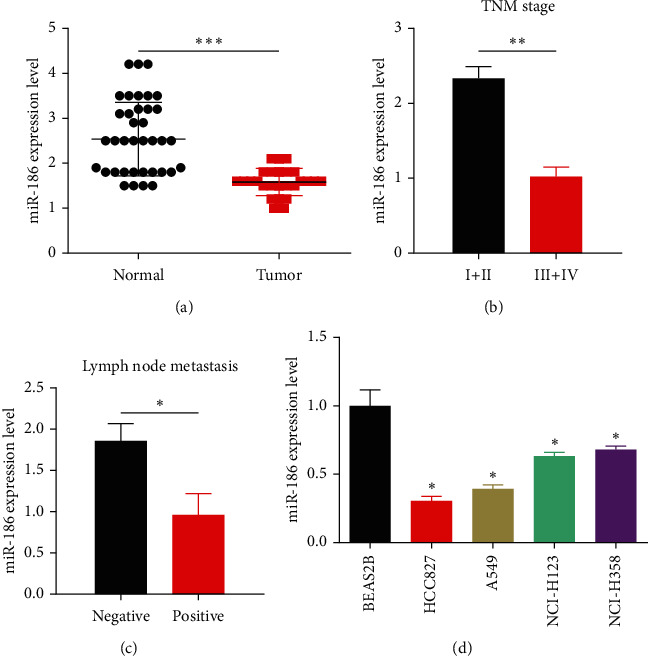
Decreased levels of miR-186 expressions in LUAD tissues and cell lines. (a) Normal tissues (*n* = 35) and LUAD tissues (*n* = 35), RT-qPCR analysis was performed to evaluate the expressions of mRNA. (b) mRNA expression of miR-186 was associated with the TNM stage of LUAD patients. (c) mRNA expression was related with lymph node metastasis. (d) mRNA expressions of miR-186 in the normal human lung epithelial cell line BEAS-2B and LUAD cell lines (HCC827, A549, NCI–H23, and NCI–H358). ^*∗*^*P* < 0.05, ^*∗∗*^*P* < 0.01, and ^*∗∗∗*^*P* < 0.001.

To demonstrate the function of miR-186 and its overexpression in the pathogenesis of LUAD, RT-qPCR was performed. Data showed a lower miR-186 expressions in LUAD tissues than normal tissues (Figure 1(a)). Furthermore, the negative correlation of the miR-186 expressions in LUAD tissues with TNM stage and lymph node metastasis was observed (Figures 1(b) and 11(c)). Additionally, in HCC827, A549, NCI–H23, and NCI–H358, the level of miR-186 was lower than that in a standard human lung epithelial cell line BEAS-2B (Figure 1(d)).

### 3.2. Effect of miR-186 on Cell Proliferation

**Figure 2:**
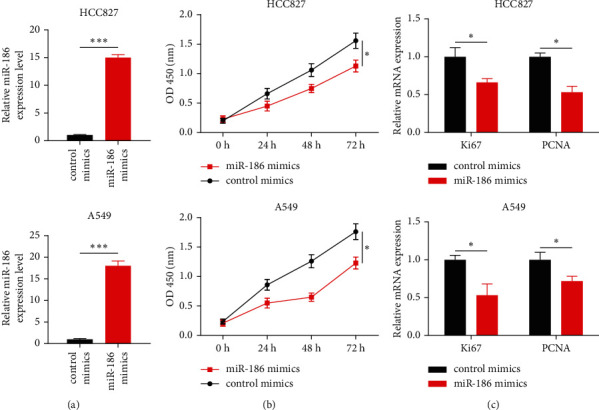
LUAD cell proliferation inhibition by overexpression of miR-186. (a) The transfected cell lines HCC827 and A549; mRNA expression was checked by RT-qPCR. (b) The HCC827 and A549 transfected cell lines. The proliferation rate was evaluated by CCK-8 assay. (c) The mRNA level of Ki67 and PCNA in HCC827 and A549 cells. *∗P* < 0.05 and *∗∗∗P* < 0.001.

In HCC827 and A549 cells, miR-186 mimics treatment significantly upregulated the miR-186 levels (Figure 2(a)). The results of the CCK-8 assay showed that overexpression of miR-186 significantly decreased the proliferation rate of HCC827 and A549 cells (Figure 2(b)). Moreover, the upregulation of miR-186 markedly reduced the levels of Ki67 and PCNA, two cell proliferation markers (Figure 2(c)). These results verify that the upregulation of miR-186 levels was able to inhibit the cell proliferation of LUAD.

### 3.3. Effect of miR-186 on Cell Migration and Invasion

**Figure 3:**
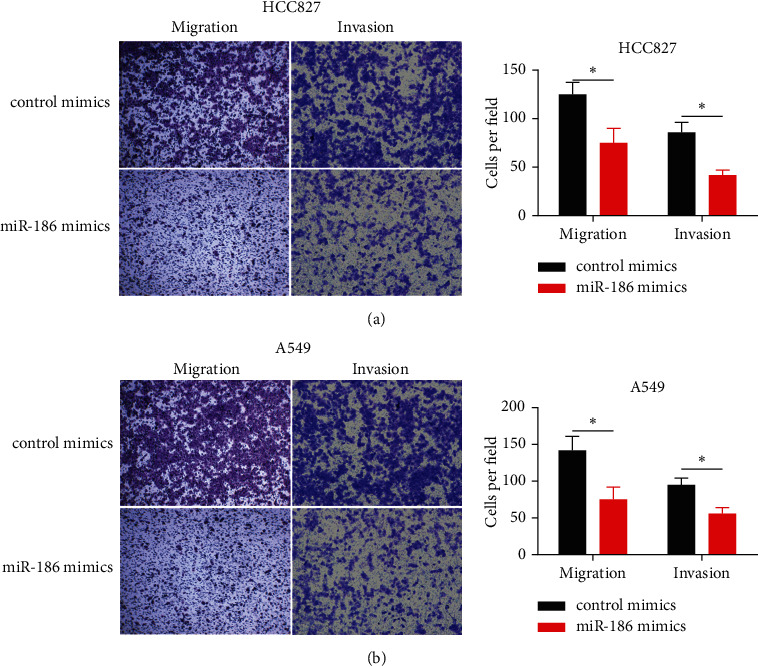
miR-186 overexpression inhibited LUAD cell migration and invasion. (a) The migration and invasion abilities of HCC827 cells transfected with miR-186 mimics or NC were estimated by transwell assay with or without the Matrigel matrix. (b) The migration and invasion abilities of A549 cells transfected with miR-186 mimics or NC were estimated by transwell assay with or without the Matrigel matrix. *∗P* < 0.05.

The function of miR-186 in regulating the cell migration and invasion potential of LUAD cells was accessed by using transwell assay. MiR-186 upregulation significantly reduced the migration and invasion abilities of HCC827 and A549 cells when compared to the control group, as shown in Figures 3(a) and 3(b).

### 3.4. Identification of Dicer1 as a Target of miR-186

**Figure 4:**
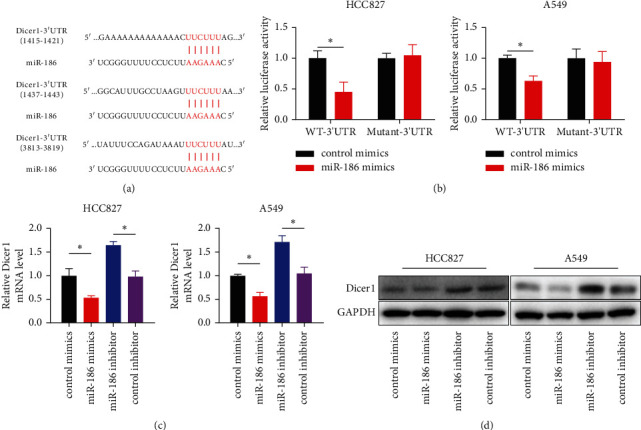
Dicer1 was targeted by miR-186. (a) Diagram of the predicted binding sites of miR-186 on the 3′-UTR of Dicer1 gene by TargetScan. (b) The luciferase activity of HCC827 and A549 cells cotransfected with miR-186 mimics, indicating wild-type or mutant 3′-UTR constructs by luciferase assay. (c, d) The mRNA examined with RT-qPCR (c) and protein detected by western blot (d) of Dicer1 in HCC827 and A549 cells transfected with miR-186 mimics, miR-186 inhibitor, or NC. *∗P* < 0.05.

TargetScan (http://www.targetscan.org), a bioinformatics algorithm, was used to predict Dicer1 as a possible target of miR-186 (Figure 4(a)). The luciferase reporter assay demonstrated that the upregulation of miR-186 considerably decreased the luciferase activity of luciferase reporter containing WT-Dicer1-3′UTR in HCC827 and A549 cells (Figure 4(b)), whereas miR-186 upregulation did not influence the luciferase activity of luciferase reporter containing mutant-Dicer1-3′UTR in HCC827 and A549 cells (Figure 4(b)). Moreover, miR-186 upregulation substantially reduced the protein and mRNA expressions of Dicer1, while a considerable increase was seen in the protein and mRNA expressions of Dicer1 by miR-186 downregulation (Figures 4(c) and 4(d)).

### 3.5. Dicer1 Was Correlated Negatively with miR-186 in LUAD Tissue

**Figure 5:**
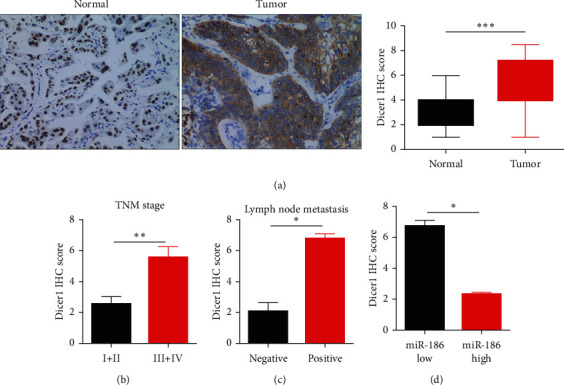
Dicer1 expression was increased in LUAD tissues and negative correlation with miR-186. (a) The Dicer1 protein level in LUAD tissues (*n* = 35) and normal tissues (*n* = 35) was stained by IHC. (b) The IHC score of Dicer1 was associated with the TNM stage of LUAD patients. (c) The IHC score of Dicer1 was associated with lymph node metastasis of LUAD patients. (d) The IHC score of Dicer1 in LUAD tissues had a negative correlation with miR-186. *∗P* < 0.05, *∗∗P* < 0.01, and *∗∗∗P* < 0.001.

The Dicer1 protein expression level was much elevated in LUAD tissues than in normal (Figure 5(a)). Additionally, the degree of Dicer1 expression in LUAD tissues was notably corrected with the TNM stage and lymph node metastasis (Figures 5(b) and 5(c)). Importantly, the protein expression level of Dicer1 in the miR-186 low expression group was higher than that in the miR-186 low expression group (Figure 5(d)).

### 3.6. miR-186 Inhibited Cell Proliferation, Migration, and Invasion via Dicer1

**Figure 6:**
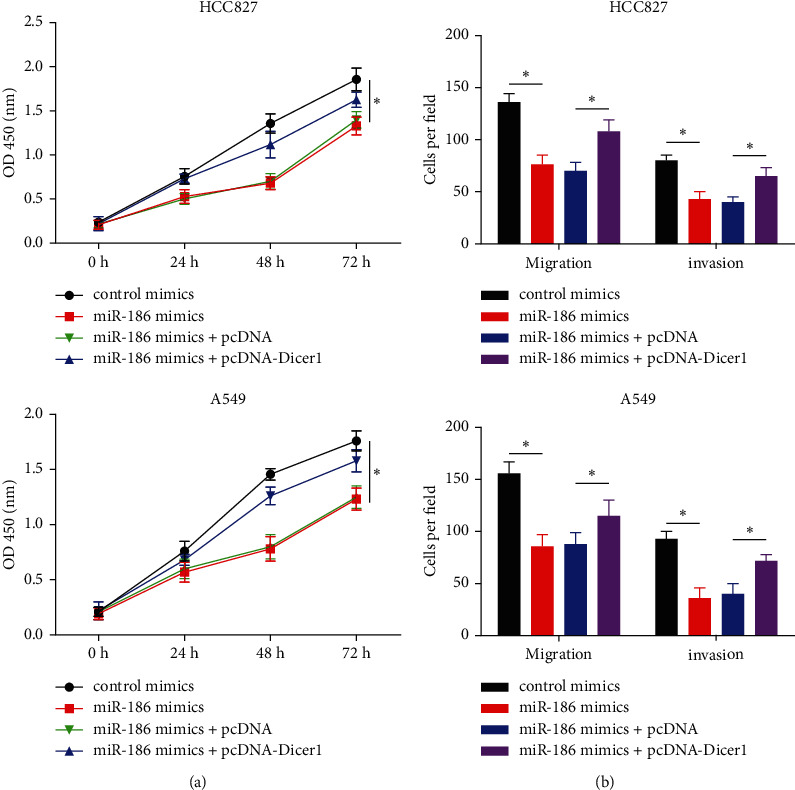
Dicer1 was involved in the effects of miR-186 on LUAD cell proliferation, migration, and invasion. (a) The proliferation rate of HCC827 and A549 cells cotransfected with miR-186 mimics and pcDNA-Dicer1 (Dicer1) was evaluated by CCK-8 assay. (b) The migration and invasion abilities of HCC827 and A549 cells cotransfected with miR-186 mimics and pcDNA-Dicer1 (Dicer1) were estimated by transwell assay with or without the Matrigel matrix. *∗P* < 0.05.

HCC827 and A549 cells were cotransfected with miR-186 mimics and pcDNA-Dicer1. The transfection with miR-186 mimics induced the growth suppression of HCC827 and A549 cells, while cotransfection with miR-186 mimics and pcDNA-Dicer1 rescued the suppression of miR-186 on cell proliferation (Figure 6(a)). Furthermore, cotransfection of HCC827 and A549 cells with miR-186 mimics and pcDNA-Dicer1 removed the inhibition of miR-186 on migratory and invasion capabilities (Figure 6(b)).

## 4. Discussion

Studies have revealed that miRNAs have been associated with tumor progression [19,20]. In oral squamous cell carcinoma, miR-146b inhibition suppressed migration, cell proliferation, and invasion via binding and downregulating HBP1 expression [10] and in the colorectal cancer miR-576-5p-targeted Wnt5a-mediated Wnt/*β*-catenin signaling pathway by inducing epithelial-to-mesenchymal transition [21]. Through targeting TrxR2 in lung adenocarcinoma cells, the miR-195-5p upregulation significantly decreased cell proliferation, migration, and invasion while increasing apoptosis [22]. A recent publication suggested that some miR displays subtype specificity in lung cancer [23]. In our study, the LUAD tissue showed a reduction in miR-186 regulation and cell proliferation migration, and invasion was suppressed by enhanced expressions of miR-186. Further investigation of the regulation and functionality of miR-186 in different subtypes of lung cancer is suggested.

In addition, miR-186 has been shown to effect many biological mechanisms in human cancer [8]. miR-186 modulated the ovarian cancer cells' cisplatin sensitivity by downregulating PIK3R3 and PTEN while enhancing the regulation of APAF1 [24]. In breast cancer cells, through modulating EREG signaling, miR-186-3p induced aerobic glycolysis and tamoxifen resistance [25]. MiR-186, which uses CDK6 as a direct target, has been shown to decrease in vivo tumor development, cell proliferation, migration, and invasion in renal cell carcinoma. In the G0/*G*1 phase, it also causes the inhibition of cell-cycle progression and induction of apoptosis [26]. miR-186-5p overexpression can suppress the colorectal cancer cell proliferation, metastasis, and epithelial-to-mesenchymal transition by targeting ZEB1 [27]. In this study, in comparison to the normal tissues, the LUAD tissues were observed with low expression of miR-186. Moreover, the expressions of miR-186 had a negative correlation with the TNM stage and lymph node metastasis. Additionally, miR-186 decreased cell proliferation, migration, and invasion by inhibiting Dicer. Our research has indicated that, in LUAD, miR-186 might have a tumor-suppressor function.

Dicer1 is a well-conserved RNaseIII endoribonuclease that contributes to microRNA biogenesis (miRNAs) [28]. Previous studies indicated that Dicer1 has a critical part in tumorigenesis and progression [29]. In colon cancer, Dicer1 impairment promoted stemness, induced an epithelial-to-mesenchymal transition, and enhanced metastatic capability of cancerous colon cells [30]. It is reported that Dicer1 was involved in miR-191/425-mediated promotion of breast cancer proliferation and metastasis [31]. The study by Ramírez-Moya et al. showed that a positive feedback loop between Dicer1 and differentiation transcription factors is essential for tumorigenesis in thyroid cancer [32]. We found that Dicer1 was an miR-186's target. miR-186 can directly reduce Dicer1 expression directly in A549, and HCC827 cells are demonstrated by the luciferase assay, RT-qPCR, and western blot assay. Moreover, Dicer1 expression levels were correlated negatively with miR-186 levels in LUAD tissue. Additionally, Dicer1 overexpression abolished the suppression of miR-186 on cell proliferation, migration, and invasion of HCC827 and A549 cells.

In summary, our data demonstrated that, in LUAD tissues, miR-186 was decreased, and its correlation with TNM stage and lymph node metastasis was found in LUAD patients. Also, cell proliferation, migration, and invasion were inhibited by the miR-186 overexpression via inhibiting Dicer 1. These findings might serve as the way for the development of novel LUAD-targeted therapies and to design novel miRNA-based therapeutic strategies against LUAD.

## Data Availability

Data will be made available on reasonable request.
